# Enhanced stochastic optimization algorithm for finding effective multi-target therapeutics

**DOI:** 10.1186/1471-2105-12-S1-S18

**Published:** 2011-02-15

**Authors:** Byung-Jun Yoon

**Affiliations:** 1 Department of Electrical and Computer Engineering, Texas A&M University, College Station, TX 77843-3128, USA

## Abstract

**Background:**

For treating a complex disease such as cancer, we need effective means to control the biological network that underlies the disease. However, biological networks are typically robust to external perturbations, making it difficult to beneficially alter the network dynamics by controlling a single target. In fact, multi-target therapeutics is often more effective compared to monotherapies, and combinatory drugs are commonly used these days for treating various diseases. A practical challenge in combination therapy is that the number of possible drug combinations increases exponentially, which makes the prediction of the optimal drug combination a difficult combinatorial optimization problem. Recently, a stochastic optimization algorithm called the Gur Game algorithm was proposed for drug optimization, which was shown to be very efficient in finding potent drug combinations.

**Results:**

In this paper, we propose a novel stochastic optimization algorithm that can be used for effective optimization of combinatory drugs. The proposed algorithm analyzes how the concentration change of a specific drug affects the overall drug response, thereby making an informed guess on how the concentration should be updated to improve the drug response. We evaluated the performance of the proposed algorithm based on various drug response functions, and compared it with the Gur Game algorithm.

**Conclusions:**

Numerical experiments clearly show that the proposed algorithm significantly outperforms the original Gur Game algorithm, in terms of reliability and efficiency. This enhanced optimization algorithm can provide an effective framework for identifying potent drug combinations that lead to optimal drug response.

## Background

Effective treatment of a complex disease, such as cancer, requires practical means to control the biological network underlying the disease. However, such therapeutic intervention is difficult in practice, due to the inherent robustness of biological networks to external perturbations and changes. Biological networks are known to be redundant at various levels, hence knocking out a specific gene or blocking a specific pathway often does not significantly change the dynamics of the network. For this reason, monotherapy using a single drug that targets a specific protein (or gene) is often limited in its therapeutic effect, and multi-target therapeutics are considered to be much more effective [1-6]. Examples of such multi-target therapeutics can be easily found in cancer chemotherapy, where most of the chemotherapy regimens consist of multiple drugs. Nowadays, combination therapies are commonly used for treating various complex diseases, including cancer and diabetes. One practical challenge in multi-target therapeutics is that the number of drug combinations increases exponentially, as the number of drugs and the number of possible concentrations increase. For example, if we want to find the optimal combination of *N* drugs, where each drug can take *L* different concentrations, there exist *L^N^* distinct drug combinations. Even for reasonably small *L* and *N*, the number of distinct combinations can be very large, making it practically impossible to find the optimal drug combination through exhaustive search. For this reason, we need a systematic method for finding the optimal combination of multiple drugs in a huge space of possible drug combinations.

Recently, a number of algorithms have been proposed for efficient prediction of optimal drug combinations [7-11]. For example, Calzolari et al. [7] proposed an optimization framework based on search algorithms that are derived from sequential decoding algorithms, widely used in digital communications [12,13]. It was shown that these search algorithms are capable of finding the optimal drug combination using only a small fraction of tests that would be needed for an exhaustive search [7]. Wong et al. [9] proposed another optimization framework based on a stochastic optimization algorithm, called the Gur Game algorithm [14,15]. In this work, Wong et al. formed a closed-loop feedback control system, where they measure the cell response to a specific drug combination, and use the Gur Game to predict a new drug combination that is likely to improve the cell response. It was demonstrated that the closed-loop optimization approach can effectively find potent drug combinations in a relatively small number of iterations [9]. Neither approaches [7,9] require any prior knowledge of the underlying biological network, which makes these methods easily applicable to various biological and medical optimization problems. As discussed in [7], if the biological system of interest shows a significantly nonlinear response to multiple drug combinations, a stochastic search algorithm, such as the Gur Game algorithm [9], is expected to perform better than nonstochastic algorithms. However, if the nonlinearity is moderate, nonstochastic search algorithms [7] may be more preferable.

An important advantage of the stochastic optimization approach is that it can effectively find potent drug combinations in the presence of multiple local optima [9]. The stochastic behavior prevents the algorithm from being trapped in a local optimum, increasing the probability of finding the globally optimal drug combination. Furthermore, stochastic algorithms can effectively cope with any uncertainty or variability that may exist in the objective function to be optimized. When our goal is to find the best combination of multiple drugs that can most beneficially affect the biological system at hand, we will have to evaluate the objective function (e.g., the desirability of the current drug combination) based on biological measurements. In such cases, measurement noise is practically unavoidable, hence the optimization algorithm should be robust to random variations that may arise from the inherent noise. From this respect, the Gur Game algorithm, which was used in [9] for predicting effective drug combinations, is especially attractive in various biological optimization problems. However, the Gur Game algorithm has also inherent limitations that may significantly degrade its overall performance for certain drug response functions. In this paper, we discuss the limitations of the original Gur Game algorithm and propose a novel stochastic optimization algorithm that can effectively address these issues. Based on various drug response functions, we demonstrate that the proposed algorithm significantly outperforms the Gur Game algorithm, in terms of both reliability and efficiency.

## Results

### Limitations of the Gur Game Algorithm

**Figure 1:**
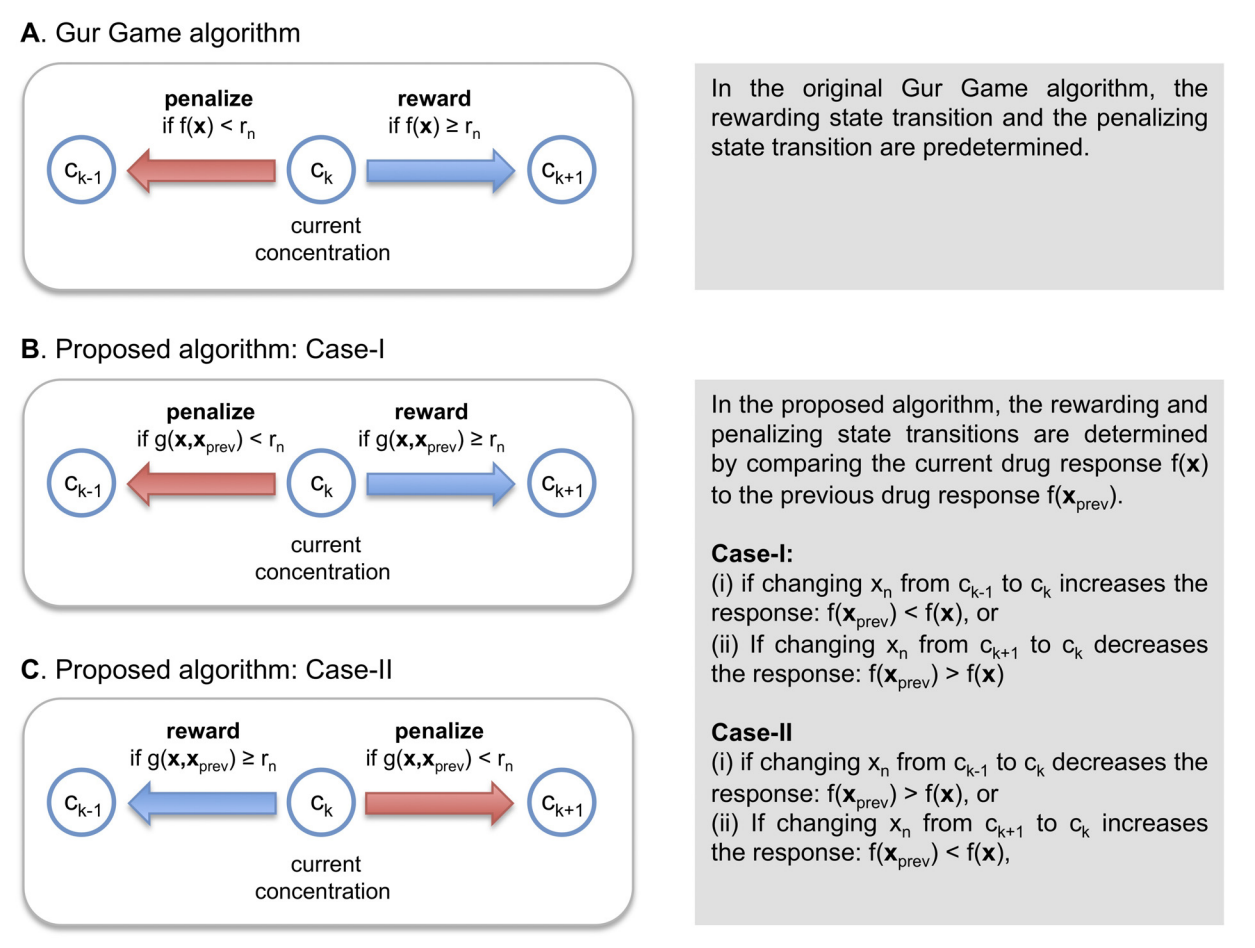
**Choosing the next state.** (A) In the Gur Game algorithm, the next drug concentration for rewarding or penalizing the current concentration is predetermined. (B,C) The proposed algorithm determines the next concentration for rewarding or penalizing the current concentration by comparing the current drug response to the previous drug response.

Let **x** = (*x*_1_, *x*_2_, ⋯ , *x_N_)* be an *N*-dimensional vector that represents the combination of *N* drugs, where *x_n_* is the concentration of the *n*th drug. We define *f*(**x**) to be the normalized drug response function that measures the desirability of a given drug combination **x**. We assume that 0 ≤ *f*(**x**) ≤ 1 for **x** ∈ **X**, where **X** is the set of all possible drug combinations under consideration. A response of *f*(**x**) = 0 implies that the combination **x** is completely ineffective, while *f*(**x**) = 1 implies that the given **x** results in the optimal therapeutic outcome. Our main goal is to find the optimal drug combination x* that maximizes the normalized drug response *f*(**x**) as follows:

Recently, Wong et. al [9] adopted a stochastic search algorithm called the Gur Game algorithm to find the most effective drug combination. They showed that the Gur Game algorithm can efficiently find potent drug combinations in a large combinatorial solution space. The basic idea of the Gur Game algorithm is to take a random walk in a finite state automaton (FSA) to find the optimal combination, where each state in the FSA represents a distinct drug combination. At each step, the normalized drug response *f*(**x**) is evaluated at the current state (i.e., for the current drug combination), based on which the algorithm randomly chooses the next state (i.e., a new drug combination) that is likely to improve the response. This is achieved as follows. The algorithm generates *N* random numbers *r_n_* ∈ [0,1] for *n* = 1, ⋯ , *N.* Each *r_n_* is compared to the current drug response *f*(**x**). If *f*(**x**) <*r_n_*, the *n*th drug is “penalized” and the concentration *x_n_* is updated accordingly. Otherwise, the *n*th drug is “rewarded” and its concentration is updated accordingly. This is illustrated in Figure 1A for the *n*th drug. Suppose the current concentration of the drug is *x_n_ = c_k_*. If the current drug response *f*(**x**) is smaller than the randomly generated number *r_n_,* the algorithm penalizes the drug by switching the concentration to *x_n_ = c_k_*_–1_. In case *f*(**x**) exceeds *r_n_,* the drug is rewarded and the algorithm switches the concentration to *x_n_ = c_k_*_+1_. Note that the direction of state transition for rewarding (or penalizing) the current drug concentration is predetermined. According to this method, the current drug concentration **x** has a higher probability of being rewarded if *f*(**x**) is high. On the contrary, the concentration **x** will be more likely to be penalized if *f*(**x**) is low. This will probabilistically drive the FSA to more desirable states that result in more effective drug responses. It should be noted that the algorithm allows a small probability of penalizing the current drug concentration even if the drug response *f*(**x**) is high (≫ 0.5). This stochastic property prevents the algorithm from being trapped in local maxima, thereby increasing the chance of finding the global maximum. In addition to this, the randomness can make the search algorithm robust against possible measurement noise in *f*(**x**). This is an important feature when our goal is to use this search algorithm in conjunction with biological experiments.

**Figure 2:**
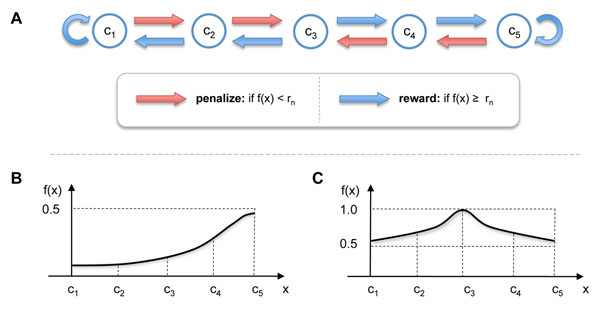
**An illustrative example.** (A) The finite state automaton used by the Gur Game algorithm. (B) An example of a drug response *f*(*x*) whose values is always below 0.5. (C) An example of a drug response whose value is always above 0.5.

Despite its many advantages, the Gur Game algorithm has also inherent limitations. To see this more clearly, let us consider the toy examples shown in Figure 2. Suppose we want to find the optimal concentration *x* of a single drug that maximizes the drug response *f*(*x*). We assume that there are five possible drug concentrations *x* ∈ {*c*_1_, *c*_2_, ⋯, *c*_5_}, hence our goal is to find the optimal concentration

among the five concentrations, using the Gur Game algorithm. For this purpose, we use the FSA shown in Figure 2A. This FSA rewards a given drug concentration *c_k_* by increasing it further if *c_k_* is higher than the central concentration (i.e., *c*_3_ in this example), unless *c_k_* is already the maximum allowed concentration. On the contrary, if the current concentration *c_k_* is lower than the central concentration, the FSA rewards it by decreasing the concentration further, unless *c_k_* is already the minimum allowed concentration. If *c_k_* is at the center, either direction is chosen with equal probability.

First of all, consider the drug response *f*(*x*) depicted in Figure 2B. As we can see, the value of *f*(*x*) is always below 0.5 and the concentration that maximizes the response is x* = *c*_5_. Since *f*(*x*) < 0.5 for every possible concentration *x*, the probability that a uniformly distributed random number *r* ∈ [0,1] will exceed *f*(*x*) is always larger than 0.5. More precisely, we have

Therefore, the state (i.e., concentration) *x* = *c*_3_ will have a higher steady-state probability compared to other states, and the random walk will visit *x* = *c*_3_ more often than the other states. As a consequence, the Gur Game algorithm probabilistically prefers the suboptimal concentration *x* = *c*_3_ over the optimal concentration *x* = *c*_5_. Next, let us assume that the drug response *f*(*x*) is as shown in Figure 2C. In this example, *f*(*x*) > 0.5 for all five drug concentrations that are under consideration, and *x* = *c*_3_ results in the maximum response. However, for the drug response in Figure 2C, we have

hence every concentration has a higher probability of being rewarded than being penalized. This probabilistically drives the FSA either to *x* = *c*_1_ or *x* = *c*_5_, both of which are suboptimal, and the optimal concentration *x* = *c*_3_ will have a lower steady-state probability compared to other concentrations. As in the previous example (Figure 2B), the Gur Game algorithm will prefer these suboptimal concentrations to the optimal concentration.

These illustrative examples clearly show that the Gur Game algorithm used in [9] may result in suboptimal performance, if the drug response *f*(**x**) is not properly normalized and/or the FSA and the directions for rewarding (and penalizing) specific drug concentrations are not properly designed. For this reason, the actual performance of the Gur Game algorithm may considerably vary depending on the underlying drug response.

### Novel stochastic search algorithm

As we discussed in the previous section, (i) proper normalization of the drug response *f*(**x***)* and (ii) effective design of the FSA is crucial for optimal performance of the Gur Game algorithm. In practice, *f*(**x***)* need to be estimated from measurements in biological experiments (e.g., through fluorescence microscopy), and normalizing *f*(**x**) such that it spans the entire dynamic range 0 ≤ *f*(**x**) ≤ 1 may be practically difficult. Furthermore, since we do not have prior knowledge of the drug response, it is very challenging to properly define the behavior of the FSA, namely, how to reward or penalize a given state, in advance.

In order to address these problems, we propose the following novel stochastic search algorithm. Let **x** = (*x*_1_*, x*_2_, ⋯ , *x_N_*) and  be the current drug combination and the previous drug combination, respectively. We assume that **x** and **x**_prev_ differ only by one element, such that  and  for some *n*. Suppose that the concentration of the *n*th drug can take a value from the set **C** = {⋯, *c_k_*_–1_, *c_k_*, *c_k_*_+1_*,* ⋯} (*c*_i_ <*c_j_* for *i* <*j*)*,* where the current concentration is *x_n_* = *c_k_*. For convenience, we define  and  such that we have , and . We assume that the FSA can make transitions from state **x** to **x***_ℓ_* and also from **x** to **x***_r_*. In the proposed algorithm, we first evaluate the function

where 0 ≤ *α* ≤ 1 is a parameter that determines the randomness of the search algorithm, which will be discussed later. It is not difficult to see that we always have *g*(**x**, **x**_prev_) ≥ 0.5. The value of the function *g*(**x**, **x**_prev_) is then compared to a uniformly distributed random number *r_n_* ∈ [0,1], based on which we decide how to make the next state transition.

First, let us consider the case when changing the concentration of the *n*th drug from  to *x_n_* = *c_k_* results in an increase of the drug response *f*(**x**_prev_) <*f*(**x**)*.* In this case, we may want to “reward” this positive change by increasing the current drug concentration further from *x_n_ = c_k_* to the next level *c_k_*_+1_. Naturally, the probability of rewarding such a positive change should be higher than the probability of penalizing it. For this reason, we increase the concentration to *x_n_ = c_k_*_+1_ if *g*(**x**, **x**_prev_) ≥ *r_n_,* and we decrease the concentration to *x_n_ = c_k_*_–1_ if *g*(**x**, **x**_prev_) <*r_n_.* In this case, we can view *g*(**x**, **x**_prev_) as the probability of rewarding the previous concentration change that improved the overall drug response. This rewarding probability will be always higher than the penalizing probability, since *g*(**x**, **x**_prev_) ≥ 0.5. Now, assume that decreasing the concentration from  to *x_n_* = c*_k_* decreases the response *f*(**x**_prev_) > *f*(**x**). In this case, we should “penalize” the decrease in *x_n_,* or equivalently, reward the increase in *x_n_.* Therefore, we again increase the concentration of the *n*th drug to *x_n_ = c_k_*_+1_ if *g*(**x**, **x**_prev_) ≥ *r_n_* and reduce it to *x_n_ = c_k_*_–1_ if *g*(**x**, **x**_prev_) <*r_n_.* This is illustrated in Figure 1B.

Next, suppose that decreasing the concentration from  to *x_n_* = c*_k_* increases the response *f*(**x**_prev_) <*f*(**x**). Since decreasing the concentration of the *n*th drug results in an improved response, we should “reward” this change by decreasing the concentration further to *x_n_ = c_k_*_–1_. Therefore, we compare *g*(**x**, **x**_prev_) to the random number *r_n_,* and we decrease the concentration from *c_k_* to *c_k_*_–1_ if *g*(**x**, **x**_prev_) ≥ *r_n_* and increase it from *c_k_* to *c_k_*_+1_ otherwise. This rule also applies when increasing the concentration from  to *x_n_ = c_k_* leads to a decrease in the response *f*(**x**_prev_) > *f*(**x**).

As we briefly mentioned before, the parameter *α* is used to control the degree of randomness in determining how the current drug concentration should be updated. If *α* = 0, we always have *g*(**x**, **x**_prev_) = 0.5, regardless of how the drug response *f*(**x**) changes for different concentrations. Therefore, when *α* = 0, the next concentration will be randomly determined between *c_k_*_–1_ and *c_k_*_+1_ with equal probability. As *α* increases from 0 to 1, we give more weight to the observed drug response change in deciding how the current concentration should be rewarded (or penalized). We can be more confident about the desirability of the predicted direction for updating the drug concentration, if the observed drug response is closer to 1 (i.e., the theoretical maximum). This is reflected by the incorporation of the term max[*f*(**x**), *f*(**x**_prev_)] in the evaluation of *g*(**x**, **x**_prev_). Unlike the original Gur Game algorithm, the proposed algorithm makes an “informed guess” on how the concentration of a given drug should be beneficially updated, by analyzing the effect of the last concentration change.

### Performance comparison: an illustrative example

**Figure 3:**
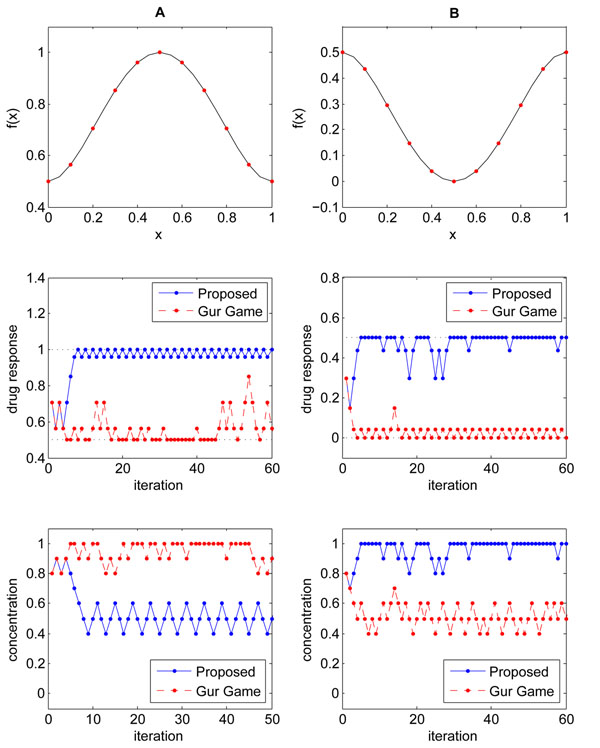
**An illustrative example.** (A) Example of a drug response function *f*(*x*) ≥ 0.5, whose maximum is located at *x* = 0.5 (top). The drug response (center) and the drug concentration (bottom) after each iteration are shown. (B) Example of a drug response function *f*(*x*) ≤ 0.5, whose maximum values are located at *x* = 0 and *x* = 1 (top). The drug response (center) and the drug concentration (bottom) after each iteration are shown.

To compare the performance between the proposed stochastic optimization algorithm and the original Gur Game algorithm [9], let us consider the two drug response functions shown in Figure 3. Figure 3A (top) shows the response *f*(*x*) to a single drug, where *x* ∈ [0,1] is the concentration of the given drug. The drug response is in the range 0.5 ≤ *f*(*x*) ≤ 1 for all *x,* and the maximum response is achieved at *x* = 0.5. As we discussed previously (see Figure 2C), such a *f*(*x*) may be problematic for the Gur Game algorithm, since the algorithm will always try to “reward” the current drug concentration as *f*(*x*) > 0.5, ∀*x*. This will drive the concentration either to the lowest concentration (*x* = 0) or the highest concentration (*x* = 1), although the maximum concentration is located at the center *x* = 0.5. Unlike the Gur Game algorithm, the performance of the proposed algorithm will not be affected, since it determines the proper way to reward (or penalize) the current drug concentration by analyzing the change in drug response that resulted from the last concentration change. In order to demonstrate the performance difference between the two algorithms, we first designed a finite state automaton with 11 states, whose structure is similar to the one illustrated in Figure 2A. The entire range of possible drug concentration *x* ∈ [0,1] was evenly divided into 11 distinct values, hence each state in the FSA corresponds to one of the following drug concentrations *x* ∈ **C** = {*c*_0_, *c*_1_, ⋯, *c*_10_}, where . Like the example shown in Figure 2A, the FSA of the Gur Game algorithm was designed such that any concentration *c_k_* that is higher than the central concentration (i.e., *c*_5_ in this case) is rewarded by increasing it further, and any *c_k_* that is lower than the central concentration is rewarded by decreasing it further. If the current concentration *c_k_* is at the center, either direction is selected with equal probability.

Figure 3A (center) shows how the drug response *f*(*x*) changes after each iteration for the two algorithms, where the blue solid line shows the drug response that results from the drug concentration predicted by the proposed algorithm. The concentration *x* predicted by the proposed algorithm after each iteration is depicted in Figure 3A (bottom), also in a blue solid line. The response and the drug concentration that are obtained using the Gur Game algorithm are respectively shown in Figure 3A (center) and Figure 3A (bottom) in red dashed lines. As we can observe in Figure 3A (bottom), the Gur Game algorithm drives the drug concentration to one of the extremes (*x* = 1 in this case), resulting in a low drug response as shown in Figure 3A (center). In comparison, we can see in Figure 3A (center) and Figure 3A (bottom) that the enhanced stochastic search algorithm can effectively find the optimal drug concentration *x* = 0.5 in just a few iterations. Note that both algorithms began from the same initial drug concentration *x*_init_ = 0.8, which was randomly selected. Another interesting fact that we can notice from Figure 3A (center) and Figure 3A (bottom) is that the proposed algorithm maintains the drug concentration around the optimal concentration *x* = 0.5, keeping the resulting drug response close to its maximum value. In fact, the steady state probability that the FSA will be at the optimal state (i.e., optimal drug concentration) is significantly higher when using the proposed algorithm compared to using the Gur Game algorithm. Similarly, the long-term average response obtained from the enhanced algorithm will be higher than the average response obtained from the Gur Game algorithm.

Figure 3B (top) shows another example drug response *f*(*x*)*,* which is always below 0.5 for all *x* ∈ [0,1], and whose maximum *f*(*x*) = 0.5 is located at *x* = 0 and *x* = 1. Based on our previous discussion (see Figure 2B), we expect that the Gur Game will always try to penalize the current drug concentration since *f*(*x*) ≤ 0.5. As a result, the algorithm will drive and keep the concentration near *x* = 0.5, resulting in a very low drug response. In fact, this can be observed in Figure 3B (center) and Figure 3B (top). Unlike the Gur Game algorithm, the proposed algorithm quickly finds the optimal concentration located at *x* = 1, and it maintains the drug response high by keeping the concentration near the optimal value. These examples clearly demonstrate the limitations of the Gur Game algorithm and also show that the proposed algorithm can effectively overcome these problems. Due to the stochastic behavior of these algorithms, the actual drug concentration predicted by the respective algorithms, as well as the resulting drug response, will be different in different experiments. However, Figures 3A and 3B are representative examples that show the typical behavior of the two algorithms.

### Predicting the optimal combination of multiple drugs

**Figure 4:**
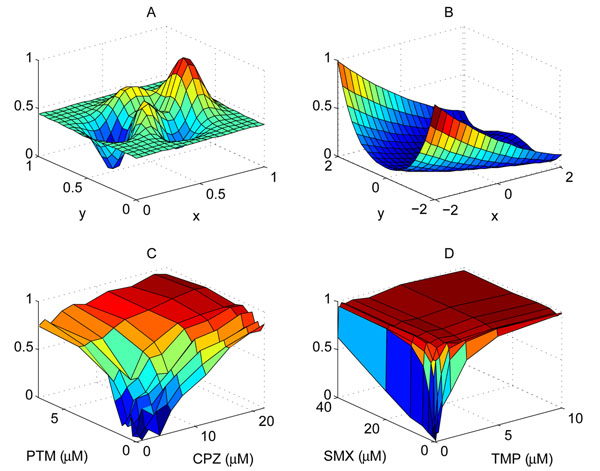
**Drug responses.** (A) Matlab peaks() function. (B) Second De Jong function. (C) Inhibition of A549 lung carcinoma cell proliferation. (D) Inhibition of the bacteria *S*. *aureus* proliferation.

To evaluate the performance of the proposed stochastic optimization algorithm, we carried out numerical simulations based on the two-dimensional response functions shown in Figure 4. The first response function *f_A_*(*x,y*) shown in Figure 4A has been obtained using the function peaks() in Matlab. In this example, we divided the interval [0, 1] into 21 evenly spaced values, hence *x*, *y* ∈ {*c*_0_, *c*_1_*,* ⋯, *c*_20_}, where  . The function *f_A_*(*x*, *y*) has been normalized so that max*_x_*_,_*_y_f_A_*(*x*, *y*) = 1 and min*_x_*_,_*_y_f_A_*(*x*, *y*) = 0. The second function *f_B_* (*x*, *y*) shown in Figure 4B has been obtained by normalizing the second De Jong function, which is defined as:

*f*(*x*, *y*) *=* 100(*x*^2^ – *y*)^2^*+* (1 – *x*)^2^,

for *x*, *y* ∈ [–2, 2]. Again, the entire range was evenly divided into 21 distinct values such that *x*, *y* ∈ {*c*_0_, *c*_1_, ⋯, *c*_20_}, where . The third response function *f_C_*(*x*, *y*) in Figure 4C shows the normalized percent inhibition of the A549 human lung carcinoma cells [16] for different combinations of chlorpromazine (CPZ) and pentamidine (PTM), which was reported in [1]. In this experiment, Borisy et al. [1] combined the antipsychotic agent chlorpromazine and the antiprotozoal agent pentamidine, and monitored the cell response to different drug combinations. For chlorpromazine, 10 different concentrations *x* ∈ {0, 1, 2, 4, 6, 8, 12, 16, 20, 22} (*µM*) had been considered. Another set of 10 concentrations *y* ∈ {0,0.25, 0.4,0.6, 0.8,1,1.5, 2,4, 6.8} (*µM*) had been considered for pentamidine, in combination with chlorpromazine. The drug response function *f_C_*(*x*, *y*) has been obtained by normalizing the percent inhibition of A549 proliferation, so that we have max_*x*, *y*_*f_C_*(*x*, *y*) = 1 and min*_x_*_,_*_y_f_C_*(*x*, *y*) *=* 0. Finally, the response function *f_D_*(*x*, *y*) in Figure 4D shows the normalized percent inhibition of the bacteria *S. aureus* for different combinations of two antibiotic drugs, sulfamethoxazole (SMX) and trimethoprim (TMP), which was reported in [6]. For trimethoprim, 9 distinct doses *x* ∈ {0,0.08, 0.16, 0.32, 0.63, 1.25, 2.5, 5, 10} (*µM*) had been tested, while a different set of 9 doses *y* ∈ {0, 0.31, 0.62, 1.25, 2.5, 5,10, 20, 40} (*µM*) had been tested for sulfamethoxazole. As before, we obtained *f_D_*(*x*, *y*) by normalizing the percent inhibition of *S. aureus* proliferation.

**Figure 5:**
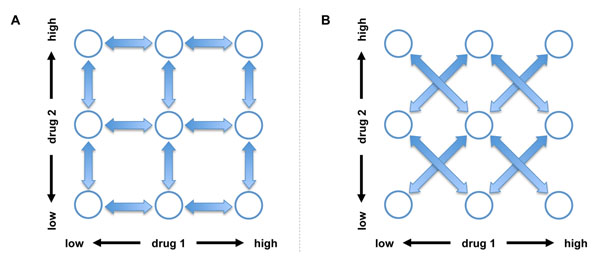
**Updating the drug concentrations.** (A) Finite state automaton for sequentially updating the two drugs. Each node corresponds to a specific drug combination. States that have the same concentration for drug 1 are aligned in the same column. Similarly, states with the same concentration for drug 2 are aligned in the same row. The arrows show the allowed transitions between sates. (B) Finite state automaton for simultaneously updating the two drugs.

For each of the four response functions shown in Figure 4, we tested the performance of the proposed algorithm as follows. First, we randomly selected the initial values of *x* and *y* (i.e., initial drug concentrations). Next, starting from the selected initial values, we used the proposed algorithm to search for the optimal drug combination (*x*, *y*) that maximizes the drug response. The parameter *α*, which is used for controlling the randomness of the search, was set to *α* = 1. In every experiment, we continued the search for *4N_x_N_y_* iterations, where *N_x_* is the number of distinct concentrations for x and *N_y_* is the number of distinct concentrations for *y.* To obtain a reliable performance estimate, this experiment was repeated 10,000 times. Based on the 10,000 independent experiments, we estimated the *success rate S,* which is defined as the relative number of experiments, in which the algorithm was able to find an effective optimal drug combination (*x*, *y*) within *N_x_N_y_* (i.e., total number of distinct drug combinations) iterations. We consider a combination (*x*, *y*) to be effective if *f*(*x*, *y*) ≥ *λ* for a given *λ* ∈ [0,1], or if the combination (*x*, *y*) is among the top *P*% combinations that result in the highest drug response. In addition to the success rate, we also estimated the average number iterations that were needed to find an effective drug combination, in case the experiment was successful. We also performed similar experiments using the Gur Game algorithm, to compare the performance of the two algorithms. Since the Gur Game algorithm does not make use of the drug response change that results from the concentration change of a specific drug, the two drug concentrations x and *y* can be either updated *simultaneously* or *sequentially* (one after the other). Sequentially updating the two drugs corresponds to using the FSA shown in Figure 5A, while updating them simultaneously corresponds to using the FSA illustrated in Figure 5B. As before, the Gur Game algorithm was designed such that it determines the direction of reward by comparing the current drug concentration to the central concentration. We evaluated the performance of the Gur Game algorithm based on the simultaneous update approach as well as the sequential update approach.

Table 1 summarizes the simulation results. First of all, we can clearly see that the proposed algorithm has a considerably higher success rate compared to the Gur Game algorithm. For the first response function *f_A_*(*x*, *y*), the success rate of the proposed algorithm was 0.95 for *λ* = 0.95, which is more than 10% higher compared to those of the Gur Game algorithm based on the two different update approaches. Furthermore, the average number of iterations that is needed by the proposed algorithm for finding an effective drug combination was only 56.6, which is much smaller than the total number of possible drug combinations *N_x_N_y_* = 441. In comparison, the Gur Game algorithm needed 82.1 iterations, when using the simultaneous update approach, and 111.7 iterations, when using the sequential update approach. The success rate for reaching the top 5% drug combinations was high for both algorithms, although the proposed algorithm had a higher success rate and required a smaller number of iterations compared to the Gur Game algorithm. The two stochastic optimization algorithms show a striking performance difference for the second drug response function *f_B_*(*x*, *y*). As we can see from Table 1, the enhanced algorithm showed 100% success rate, while the Gur Game algorithm was able to find an effective combination only 9% of the time for *λ* = 0.95, and only 12% of the time for *P* = 5%. The number of iterations that was needed by the proposed algorithm to find an effective combination was only 56.7 for *λ* = 0.95 (and only 49.5 for *P* = 5%), which is again significantly smaller than the total number of combinations *N_x_N_y_* = 441. For this drug response, the Gur Game algorithm needed less number of iterations for finding an effective drug combination, *if* the search was successful. But it has to be noted that the Gur Game algorithm was not able to find an effective combination most of the time.

**Table 1:** Performance of the proposed stochastic optimization algorithm.

		Gur Game algorithm (simultaneous)		Gur Game algorithm (sequential)		Proposed algorithm	

		success rate	# of iterations	success rate	# of iterations	success rate	# of iterations
PEAKS	*λ* = 0.95	0.84	82.1	0.82	111.7	**0.95**	56.6
	*P* = 5%	0.96	29.6	0.97	37.9	**1.00**	20.6

DE JONG (2ND)	*λ* = 0.95	0.09	43.1	0.09	39.9	**1.00**	56.7
	*P* = 5%	0.12	28.6	0.12	30.1	**1.00**	49.5

CANCER	*λ* = 0.95	0.63	16.3	0.62	18.8	**0.94**	13.1
	*P* = 10%	0.68	15.1	0.64	18.2	**0.95**	12.6

BACTERIAL	*λ* = 0.95	0.91	4.0	0.90	5.0	**0.99**	4.6
	*P* = 10%	0.71	7.2	0.73	9.6	**0.75**	7.6

For the response function *f_C_*(*x*, *y*) in Figure 4C, which shows the normalized human lung carcinoma percent inhibition for different combinations of pentamidine and chlorpromazine, the proposed algorithm yielded a success rate of 0.94 for *λ* = 0.95 and 0.95 for *P* = 10%. In both cases, the success rate was around 30% higher compared to that of the Gur Game algorithm, as we can see in Table 1. The proposed optimization algorithm needed only 13.1 iterations for finding an effective drug combination for *λ* = 0.95 and 12.6 iterations for *P* = 10%. These numbers are significantly smaller than the total number of drug combinations *(N_x_N_y_* = 100), and also smaller than the number of iterations needed by the Gur Game algorithm.

Finally, the proposed algorithm also yielded a higher success rate than the Gur Game algorithm for the normalized bacterial response *f_D_*(*x*, *y*) shown n Figure 4D. On average, both algorithms required only about 4∼10 iterations for finding an effective drug combination, which is much smaller compared to the total number of possible combinations *(N_x_N_y_* = 81). One thing we can notice is that the proposed algorithm had a success rate of 0.75 for *P* = 10%, which is lower than its success rate in other experiments. This is mainly because the drug response function *f_D_*(*x*, *y*) has a large number of drug combinations (*x*, *y*) that yield high drug response. In fact, the top 10% drug combinations yield a very high drug response of *f_D_*(*x*, *y*) ≥ 0.99, and finding such combinations is especially challenging due to the plateau-like shape of the response *f_D_*(*x*, *y*).

## Conclusions

In this paper, we proposed a novel stochastic optimization algorithm that can efficiently find optimal drug combinations. The proposed algorithm extends the Gur Game algorithm [9] by incorporating additional information about how the concentration change of a specific drug affects the overall drug response. By comparing the drug responses to two different drug combinations, which differ only in the concentration of a single drug, the new algorithm determines how to update the current concentration of the given drug to improve the response. In this way, the algorithm can adapt itself to the underlying drug response function, which is not known in advance. As a result, although the new algorithm still does not require any prior knowledge on how the the biological system of interest (e.g., cancer cell) responds to different drug combinations, it consistently outperforms the Gur Game algorithm for various types drug response functions. Simulation results show that the novel optimization algorithm can find effective drug combinations more reliably and also more efficiently, compared to the Gur Game algorithm. Unlike the Gur Game algorithm, the new algorithm is not very sensitive to different (and possibly suboptimal) normalizations of the drug response. Furthermore, the stochasticity of the algorithm is useful in handling any uncertainty (or variability) that may be present in the drug response function. Since such variability is typical when we have to evaluate the drug response function *f*(**x**) from biological measurements, this stochastic property is practically important when using the algorithm in conjunction with biological experiments, as in [9]. Although we have mainly applied the proposed algorithm for optimizing two drugs, it can be directly used for optimizing the concentrations of multiple drugs in a straightforward manner.

## Authors' contributions

BJY developed the algorithm, performed the numerical experiments, and wrote the paper.

## Competing Interests

The authors declare that they have no competing interests.
